# Paraneoplastic neurological complications of breast cancer

**DOI:** 10.1186/s40164-016-0058-x

**Published:** 2016-10-24

**Authors:** Ibrahim Fanous, Patrick Dillon

**Affiliations:** 1 University of Virginia, Charlottesville, USA; 2 UVA Division of Hematology/Oncology, UVA, Box 800716, Charlottesville, VA 22908 USA

**Keywords:** Breast cancer, Epidemiology, Paraneoplastic, Cerebellum, Stiffness, Retinopathy, Encephalitis

## Abstract

Breast cancer is the most frequent cause of cancer of women in much of the world. In countries with screening programs, breast cancer is often detected before clinical symptoms are apparent, but occasionally the occurrence of a paraneoplastic syndrome precedes the identification of cancer. In breast cancer, there are known to be paraneoplastic endocrine syndromes and neurologic syndromes. The neurologic syndromes are often hard to identify and treat. The neurologic syndromes associated with breast cancer include cerebellar degeneration, sensorimotor neuropathy, retinopathy, stiff-persons syndrome, encephalitis, and opsoclonus–myoclonus. Most of these are mediated by antibodies against known neural antigens, although some cases appear to be mediated by non-humoral mechanisms. Treatments differ depending upon the syndrome type and etiology. Outcomes also vary depending upon duration of disease, the treatments used and the responsiveness of the underlying cancer. A thorough review of the published literature is provided along with recommendations for management and future research.

## Background

Breast cancer is the most common non-skin cancer in women in the United States with an estimated one in eight lifetime risk. Similarly high rates are observed in many other parts of the world. The incidence rate in the United States has remained stable for the last 15 years. Breast cancer death rates, however, decreased by 34 % since 1990 suggesting that progress is being made in breast cancer treatment and detection. Nevertheless, a cure for metastatic disease has not been achieved and some deficiencies in treatment of advanced disease persist [1, 2]. One important deficiency in the field of breast cancer is a lack of robust data for the characterization, detection and management of paraneoplastic neurologic syndromes. Since paraneoplastic syndromes are rare in breast cancer, no prospective studies have been performed. Paraneoplastic neurologic syndromes are often severely debilitating, hard to diagnose, and challenging to treat.

It is unknown whether certain patient subsets might be at greater risk for paraneoplastic syndromes in breast cancer. Receptor typing and histological subtype have little known impact on the likelihood of developing paraneoplastic syndromes. Other clinical factors such as lymph node involvement, lymphovascular space invasion and perineural invasion are known adverse factors for breast cancer in general, but it is unknown whether these factors confer increased risk for development of paraneoplastic syndromes. Finally, nodal status is related to rate and timing of metastatic disease normally [3], but has no relationship to timing of paraneoplastic syndromes. Those syndromes may develop at any point in the course of breast cancer, even preceding the formal diagnosis of breast cancer in some cases [4].

The diagnosis of a paraneoplastic syndrome is challenging due to heterogeneity in timing, symptomatology and presence of onconeural antibodies. Antibodies are only found in 60–70 % of paraneoplastic syndrome patients with breast cancer. Therefore antibody testing may be helpful if positive, but the absence of antibodies cannot rule out a paraneoplastic neurologic syndrome. To address these issues, the international panel of neurologists defined four components for the diagnosis of paraneoplastic neurological disorders. These are, (1) the presence of neurological symptoms, (2) a diagnosis of cancer within 4 years from the onset of the neurological manifestations, (3) exclusion of other neurological disorders and (4) at least one of the following: CSF analysis showing inflammation with negative cytology, a brain MRI demonstrating a lesion in the temporal lobe, or the finding of epileptic activity in the temporal lobes by electroencephalogram (EEG) [5–7].

**Fig. 1:**
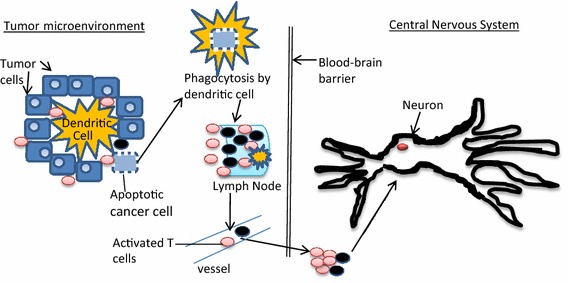
Simplified mechanism of paraneoplastic immune neurologic injury. This figure summarizes the theorized immune mechanism of paraneoplastic neurologic syndromes. The beast tumor microenvironment contains immune cells such as CD4^+^ T cells, CD8^+^ T cells, NK cells, macrophages, dendritic cells (DC) and others. When tumor cells undergo apoptosis, the DC’s may phagocytose them, travel to lymphoid nodes (or other lymphoid structures) to present antigen to CD4^+^ and CD8^+^ T-cells and even B-cells. Certain activated T cells and autoimmune antibodies may then cross the blood brain barrier where normally immunologically privileged neurons may be targeted by antibody or T cells or both

It is well documented that breast cancer is immunogenic and several recent successes with checkpoint inhibitors and vaccines attest to this fact [8]. Indeed several unique and shared tumor antigens have been identified for each subtype of breast cancer. The TCGA studies have confirmed that mutations or loss of the p53 tumor suppressor are common and may contribute to the cancer phenotype. By permitting unchecked cell division, these p53 defects likely are permissive to expression of mutated or mis-folded proteins that would otherwise not be “visible” to the immune system. Inherited and acquired defects in DNA repair (BRCA 1&2, PALB2, CHEK2, etc.) are also well known contributors to mutation in breast cancer. There are many other cellular events and mechanisms which contribute to aberrant antigen expression in cancer (DNA methylation/acetylation changes, mRNA binding protein changes, exosomes, etc.) The ultimate result of several sub-cellular changes in the cancer cell and the stroma is that the immune system is frequently primed to recognize tumor-associated epitopes as foreign. The resulting aberrant antigen/neo-antigen expression, glycosylation, and changes in protein degradation occur in the setting of cancer cells that are undergoing rapid expansion which sets the stage for dendritic cells to scavenge cellular debris and carry “hidden” or otherwise novel antigens to the draining lymph nodes and other lymphoid organs. In the nodes or lymphoid organs, amplification of the immune response may occur including B and T lymphocyte selection and expansion [9–12]. Unfortunately not all the B and T lymphocyte responses produce anti-tumor effects. Some may be tolerogenic, some may be involved in memory response, and rarely some may result in responses against auto-antigens. Auto-recognition then leads to autoimmune and/or paraneoplastic syndromes. Additional T cell and B-cell mediated mechanisms of neuronal damage have been well described previously and are summarized in Fig. 1.

Paraneoplastic neurological syndromes have been reported in breast cancer as early as 1968. Those syndromes can be quite debilitating when they occur. Currently, breast cancer related neurologic paraneoplastic syndromes include sensory and motor-type neuropathies, paraneoplastic cerebellar degeneration, opsiclonus–myoclonus syndrome, stiff person syndrome, enchephalomyelitis (including limbic encephalopathy) and paraneoplastic retinopathy.

The most compelling study of pathobiology was reported for 131 breast cancer patients in 2011. In that study, it was suggested that the tumor microenvironment and bone marrow resident T cells may play a role in the development of humoral paraneoplastic syndromes [13]. Specifically, T cell reactivity to autoantigens was tested by ELIspot assay. Patients with autoreactive T cells had intratumoral IFN-α concentrations above 10 pg/ml and patients with serum autoantibodies had significantly increased levels of intratumoral IL-12 compared to antibody negative patients (p = 0.04). Serum samples from breast cancer patients were examined for anti-nuclear antibodies (ANA), anti-neutrophil cytoplasmic antibodies (ANCA), thyroid-stimulating hormone receptor antibodies (TRAb), rheumatoid factors (RhF), and anti-thyreoglobulin antibodies (TgAb). ANAs were detected in 29/85 (34.1 %), ANCAs in 3/86 (3.5 %), TRAbs in 0/86 (0 %), RhF in 4/85 (4.7 %), and TgAbs in 27/85 (31.8 %) patients analyzed. In total, 49 out of 86 patients (57 %) had at least one positive antibody result. The same group had previously detected reactivity against HLA-A^*0201^-restricted tumor-associated antigens in 33 % of all breast cancer patients versus reactivity against normal breast tissue-associated antigens was seen in 24 % [14]. Further, IFN-α is increased in breast cancer patients with immune recognition of tumor. Since IFN-α amplifies T-cell autoreactivity by supporting T-cell activation and survival, it likely contributes to paraneoplastic syndromes. Likewise, IFN-α suppresses the generation of CD4^+^FoxP3^HI^ regulatory T cells, thus weighing immune homeostasis toward autoimmunity [13, 14].

## Paraneoplastic cerebellar degeneration

Paraneoplastic cerebellar degeneration (PCD) is fairly rare in general and is seen in less than 1 % of cancers. It has been associated with small cell carcinoma of the lung, Hodgkin’s lymphoma, breast cancer and gynecologic malignancies. It presents clinically with moderate to severe truncal ataxia, nystagmus, vertigo, dysarthria and sometimes diplopia. The onset may be acute or subacute and by definition should be without the presence of brain metastases or direct CNS invasion [15].

The first case to be diagnosed with PCD was attributed to Brouwer whose patient suffered from “pleomorphic cell sarcoma of the pelvis”. Another case of PCD was reported in 1929 occurring 2 years after a mastectomy. A total of 62 cases of PCD were eventually identified and fully characterized between 1966 and 1990. Those 62 patients tended to present with early stage cancers. Only three of them were described to have had metastatic disease at the time of the PCD diagnosis. Two additional patients were described as having only regional nodal disease. Cerebrospinal fluid (CSF) analysis was performed and showed the presence of the anti-Yo antibody (previously termed anti-Purkinje antibody) in 19 patients. All 19 patients with anti-Yo positivity in the CSF also had anti-Yo positivity in their sera. At autopsy, PCD patients show decreased cerebellar size, diffuse loss of Purkinje cells throughout the cerebellar cortex, and also a loss of granule and basket cells.

The clinical presentation of PCD is variable. Trunk and limb ataxia, dysarthria, diplopia and vertigo tend to predominate in the anti-Yo mediated cases while the frequency of nystagmus and dysarthria is lower in patients mediated by anti-Ri (antineuronal nuclear antibody type 2) or other antibodies discussed below. Psychiatric symptoms have also variably been reported. In anti-Hu associated PCD, non-cerebellar symptoms and psychiatric symptoms were more frequent. It is estimated that 20 % of patients with PCD will develop cognitive disorders [16].

A clinical challenge for PCD diagnosis is that the cerebellum is radiographically normal in most of the reported cases of PCD and many patients may present well before a cancer diagnosis is known. Indeed, radiographic changes in cerebellar size are usually only seen in the late stages of the disease. Most of the time, PCD patients have associated onco-neural antibodies, most often anti-Yo or anti-Ri which can help confirm the diagnosis. In the rare antibody negative PCD cases, the neurologic disease appears to be rapidly progressive and unrelenting [17–19]. The neurologic manifestations of PCD often ante-date the diagnosis of breast cancer. Indeed, a case series observed neurologic manifestations of PCD before breast cancer diagnosis in 77 % of cases. The time range in that series was 2–41 months before cancer diagnosis [18].

A majority of PCD cases are mediated by anti-Yo antibodies. In 1987 the target proteins of anti-Yo were found. The proteins are coded by the cerebellar degeneration related gene (CDR) which is expressed mainly in the Purkinje cells. There are two known isoforms, a minor 34-kD antigen (CDR34) and a 64-kD antigen (CDR64) [20, 21]. Not surprisingly, the antibodies usually react against autologous tumor cells expressing these antigens (aberrantly). Antibody dependent cellular cytotoxicity has also been reported, but there remains some debate as to whether the anti-Yo is the primary mediator of the cellular damage or merely a bystander. To further complicate the debate about the direct role of anti-Yo, it was found that anti-Yo may be found in healthy non-cancer patients and may be associated with attention deficit disorder. Thus, it remains unclear what role anti-Yo plays in cerebellar Purkinje cell loss [22].

Despite the debate about mechanism, the finding of anti-Yo in any patient with cerebellar disease should be a red flag to look for underlying malignancy. In one series, 100 % of anti-Yo patients were diagnosed with malignancy on further follow-up [23]. On the other hand, not all cases of PCD are associated with anti-Yo specifically. In a series of 50 patients with antibody associated PCD, 19 patients had anti-Yo, 16 had anti-Hu, seven had anti-Tr, six had anti-Ri and two had anti-mGluR1 (Table 1). Ultimately all of patients that tested positive for anti-Yo, anti-Tr and anti-mGluR1, had a clinical syndrome consistent with PCD. Another series of 92 patients with paraneoplastic neurologic syndromes associated with breast or gynecologic cancers found 63 patients with onconeural antibodies (50 anti-Yo, 5 anti-Hu, 5 anti-Ri, and 3 anti-amphiphysin). Fifty-seven patients (62 %) developed cerebellar ataxia; 88 % of them were found to have anti-Yo [24].

**Table 1:** Summary of anti-neuronal antibodies in paraneoplastic neurologic diseases

	Primary anti-neuronal antibody	Other associated anti-neuronal antibodies
Paraneoplastic cerebellar degeneration	Anti-Yo [16–21]	Anti-Hu Anti-Tr Anti-Ri Anti-mGluR1 [22, 23]
Paraneoplastic retinopathy	Anti-enolase [25–28]	Anti-Recoverin Anti-Transducin Anti-Carbonic AnhydraseII Anti-Arrestin β [29, 30]
Opsiclonus–myoclonus syndrome	Anti-Ri [36, 62]	Anti-Yo Anti-Hu Anti-Amphiphysin Anti-Nova-1 Anti-Nova-2 [31–33]
Stiff person syndrome	Anti-GAD and Anti-amphiphysin [34, 35]	
Paraneoplastic sensory peripheral neuropathy	Anti-Hu, Anti-Yo, Anti-Ri [36–38]	
Paraneoplastic limbic encephalomyelitis	Anti-Hu [39–44]	Anti-Ta Anti-Ma [39–44]

PCD has a poor prognosis and treatment options are limited. The use of intravenous immunoglobulin (IVIG), corticosteroids, plasmapheresis and cyclophosphamide, have all been tried individually or in combination, and they have low response rates. Because PCD has an autoimmune mechanism of action, elimination of the antigen source may be helpful. Indeed, resection of the primary tumor is reported to reduce the neurological manifestations of PCD [45, 46]. The median survival time in patients treated with anti-tumor therapy is longer than those not receiving anti-tumor therapy regardless of the use of immunotherapy (hazard ratio 0.3; 95 % confidence interval 0.1–0.6; p = 0.004) [47]. Despite a survival advantage to treatment of the underlying cancer, the functional outcomes for PCD patients remain abysmal. In a series of 55 women with breast and gynecologic cancer and anti-Yo mediated PCD, the most common neurologic outcome was severe disability. PCD was disabling in 37 of the patients to the degree that they were unable to walk or sit unsupported. These late disabling neurological manifestations generally did not respond to treatment [16].

The long-term outcome was further evaluated in a series of 34 women with PCD. Twelve of the patients had breast cancer. The PCD diagnosis preceded the diagnosis of primary tumor in 19 patients. Six patients were diagnosed with PCD after diagnosis and treatment were complete. Overall, the cause of death was related to tumor progression in 12 patients and to PCD in 9 patients and unknown in two patients. The median survival time was 22 months in patients with ovarian cancer and 100 months in patients with breast cancer. At the time of analysis, 13 out of the 18 patients with ovarian cancers were dead (72 %) and cancer progression was the cause of death in 61 %. By contrast only 5 out of the 12 patients with breast cancer were dead (42 %) [48, 49].

In terms of treatment for PCD, there is one patient series describing use of a combination of IVIG, cyclophosphamide and methylprednisolone. The patients received one to nine cycles (mean 3.5) of that combination. There was no reported toxicity and the tolerance was satisfactory. Unfortunately, the best response to treatment was stable disease, which was observed in three out of seven patients [50]. Another descriptive report of 15 cases of antibody-mediated PCD analyzed the use of IVIG either alone or in combination with other therapies. It was concluded that the best responses were seen when IVIG treatment was begun within the first month of symptoms. Patients that received treatment between 1 and 3 months of symptom onset often had stable disease, while patients who received therapy beyond three months from onset had poorer outcomes. This series was unable to compare between double and triple agent therapy. It was suggested that high doses of IVIG and steroids as early as possible in the course of PCD may be useful [51].

## Retinopathy

Paraneoplastic retinopathy is a rare breast cancer complication, which like other paraneoplastic syndromes is mediated by aberrant immune cross-recognition of antigen. Affected patients experience photosensitivity, difficulties in visual acuity, impaired color vision, peripheral scotomas or prolonged dark adaption. Paraneoplastic retinopathy is most associated with melanoma and small cell lung cancer but it has also been reported with breast cancer and less commonly with other cancers (Table 2) [52, 53]. Indeed in one academic retina clinic, breast cancer made up 31 % of cancer associated retinopathy cases [54]. Because the retina is immunologically privileged, the immune system is not normally exposed or tolerized to the retinal antigens and thus aberrant expression by cancer cells may result in B and T cell recognition of auto-antigens. It is known that abnormal expression of retinal proteins occurs in breast cancer cells and presumably that is the source for dendritic cell exposure to retinal antigen and the eventual B-cell production of autoantibody [55–58]. The production of anti-retinal antibodies is shown to cause direct damage to retinal enolase, rods, cones, and several other retinal structures [59–63].

**Table 2:** Lists the paraneoplastic retinopathy-associated antigens and their associated primary cancers

Type of cancer	The associated antigens
Breast cancer	Enolase, Recoverin, Transducin β
Gynecological cancers	Enolase, Aldolase C, Carbonic anhydrase II, Recoverin and GAPDH
Small cell lung cancer	Recoverin
Non-small cell lung carcinoma	Transducin β, Carbonic anhydrase II
Colon adenocarcinoma	Transducin β, Recoverin, Carbonic anhydrase II
Bladder adenocarcinoma	Enolase
Skin melanoma	Enolase, Transducin β, Arrestin
Skin squamous cell carcinoma	Recoverin
Cutaneous B cell lymphoma	Enolase
Prostate cancer	Enolase, Carbonic anhydrase II

The molecular weights for target antigens are: Enolase (23 kDa), Recoverin (46 kDa), transducin β (40 kDa), carbonic anhydrase II (30 kDa) [26, 54, 64, 65]

As with paraneoplastic cerebellar degeneration previously discussed, there are occasional retinopathy cases for which antibody cannot be detected in serum. It remains unclear whether the failure to detect antibody in some patients is a technical limitation or truly due to alternative mechanisms of retinal injury (i.e. T-cell mediated). Nevertheless, we do know that seropositive patients have a worse prognosis than seronegative patients. Also, abnormalities in the rod and cone photoreceptor function, as confirmed by electroretinogram, were seen three times more frequently in seropositive patients. Finally, central vision loss was more evident and more frequent in seropositive patients [25–28].

Retinal antibodies often develop prior to the clinical diagnosis of breast cancer. The most common of these antibodies are anti-enolase which was found to be the most prevalent antibody (32 %) in breast cancer related retinopathy. Anti-recoverin was found in 4.5 % of retinopathy patients between one and 15 years after diagnosis of breast cancer [29, 30]. Since the titer of anti-retinal antibody is correlated with disease progression, it may be used as a surrogate marker of disease activity [64, 66, 67]. Other cancer associated retinal autoantibodies have been described (Table 2), most of which have been shown to induce apoptotic death of retinal cells in vitro [68–72].

The actual titer may be more important than the mere presence of autoantibody when dealing with retinopathy. Since healthy patients sometimes are found to have very low titers of retinal auto-antibodies, a few studies have examined the impact of titer on visual manifestation and found a link. Most notably, a study of 178 patients with lung or breast cancer was reported. In the study, a high anti-enolase titer was most associated with late stage cancer and with greater damage compared to low titer [73–75]. In patients with high titers, ring scotomas, retinal artery narrowing, and reduced response on electroretinogram were reported [31, 76, 77]. It is notable that clinical differences have been suggested with different auto-antibodies. For example, anti-enolase patients show rapidly progressive loss of vision while anti-recoverin patients have slowly progressive loss of vision and carbonic anhydrase II antibody patients tend to have color vision loss.

The predominant study of paraneoplastic retinopathy associated with breast cancer involved 295 patients. Serum samples from patients were examined and six patients were found to have high titers of retinal auto-antibodies. On complete ophthalmologic examination, all of the patients had normal intraocular pressures. None of the patients showed any abnormalities in ophthalmoscopic, visual field or electroretinogram exams. All six had abnormal visual evoked potentials. The most frequent antibody was anti-enolase followed by anti-arrestin. The peripheral blood lymphocytes of two of the six patients responded to retinal antigens, thus further suggesting that T cells cooperate with antibody in causing retinal damage in cancer associated retinopathy [31, 32].

## Paraneoplastic opsoclonus–myoclonus syndrome

Paraneoplastic opsoclonus–myoclonus syndrome (OMS) has been reported as one of the paraneoplastic neurological manifestations of breast cancer [33]. It is usually associated with anti-Ri antibodies that cross-react with two antigens, Nova-1 and Nova-2 which are widely expressed in the CNS. Other auto-antibodies have been also associated with OMS, such as anti-Yo and anti-Hu [78]. The syndrome is typically characterized by rapid, involuntary, multivectoral, conjugate fast eye movements (opsoclonus) and brief, involuntary twitching of muscles (myoclonus) with or without accompanying ataxia, aphasia, strabismus or mutism. Historically it is most often associated with neuroblastoma.

Since adult paraneoplastic OMS is rare, there are only small case series and a few case reports associating it with breast cancer. An early case series of 14 patients observed the syndrome in small-cell lung cancer (SCLC) (nine patients), non-SCLC (one patient), breast carcinoma (two patients), gastric adenocarcinoma (one patient) and renal cell carcinoma (one patient). There was frequent encephalopathy in these paraneoplastic OMS patients. Serum from two of the 14 paraneoplastic OMS patients contained anti-neuronal antibodies and one was a patient with underlying breast cancer (anti-Ri in that case). The paraneoplastic OMS patients had steadily declining clinical courses despite treatment with corticosteroids and/or IVIG. OMS was the cause of death in five of the 14 patients. The authors concluded that paraneoplastic OMS tended to occur later than idiopathic OMS and had a worse outcome [34, 35].

A later series of twenty-one patients from the Mayo clinic with OMS were studied. In these 21 OMS patients (median age of 47 years), underlying cancer was detected in only three patients (two patients with breast cancer and one patient with small cell lung cancer). An infectious etiology was presumed in the rest of the study population. Sixteen of 21 patients were given systemic therapy and immunotherapy. One patient received clonazepam alone and two received immunotherapy alone. Of the 19 treated patients, 13 patients had remission, three patients improved and the other three patients died. Likewise in a literature review of OMS, the cause of death was of neurologic complication in 60 of 116 patients. Seven patients in that series had breast cancer. The most common antibody was anti-Ri seen in 15 patients. Our literature review concludes that immunotherapy treatment may lead to partial or complete recovery of OMS in some cases [79].

## Stiff-person syndrome

Stiff-person syndrome (SPS) was previously-known as stiff-man syndrome and has been reported as one of the paraneoplastic neurological manifestations of breast cancer. It is an uncommon auto-immune disease that usually presents with severe muscle spasms in addition to thoracolumbar stiffness. It is commonly associated with elevated levels of glutamic acid decarboxylase (GAD) antibodies, an auto-antibody often seen in diabetes mellitus type I. A second variant is reported and is associated with anti-amphiphysin antibodies [80, 81]. A SPS project from Yale has examined the differences in clinical presentation between the two types of SPS disease. Sera of 845 patients with any stiffness complaints were studied and a total of 621 patients were clinically suspected of having SPS. A total of 116 patients had GAD antibodies and 11 patients had amphiphysin antibodies in association with a clinical stiffness syndrome. In the study, all the anti-amphiphysin SPS patients were female and 10 of them had underlying breast cancer. Only one patient in the anti-GAD group had underlying breast cancer. The patients in the anti-amphiphysin group were older than patients in the anti-GAD group (mean age of 58 vs 44 years, p = 0.002). Patients in both groups showed stiffness, and substantial pain. None of the patients in the anti-amphiphysin group had diabetes mellitus. Eight of the 11 anti-amphiphysin patients had an EMG showing continuous unit activity of affected muscles. Regarding treatment of stiffness in the anti-amphiphysin group, nine patients were reported to have a good response to high dose benzodiazepine (>50 mg/day); four patients were steroid responsive; and three patients had a dramatic improvement after cancer excision/chemotherapy. Unfortunately, one patient did not have any response to treatment and died. The anti-amphiphysin patients did not show any benefit from IVIG use [81–83]. Most of the anti-GAD patients were reported to have diabetes. They were also reported to be responsive to high doses of benzodiazepine and to IVIG. The pattern of stiffness was different between the two groups. The anti-GAD patients showed more stiffness in the legs and spine than abdomen, arms, and neck. For anti-amphiphysin patients, the pattern was equal involvement of the above areas (p < 0.001) [84, 85].

The target antigen for anti-amphiphysin has been described as a 128-kD brain protein found at synapses [86]. The amphiphysin antibodies have primarily been associated with invasive ductal carcinoma of the breast. In one report amphiphysin antibodies were not detected in SPS patients without underlying breast cancer nor were they found in the general cancer population [87].

Treatment of SPS may include benzodiazepines and/or IVIG depending on the antibody type. The anti-amphiphysin patients may also respond to plasmapheresis, steroids and treatment of the primary tumor [82, 88]. Older reports suggests that anti-GAD patients respond to diazepam, baclofen and steroids [89].

Other SPS cases in the literature include a breast cancer patient found to have both anti-GAD and anti-amphiphysin antibodies. The patient was reported to improve here SPS symptoms after surgery and corticosteroids and she was reported to be stable for four years on anti-estrogen treatment [90]. Another patient with breast cancer was reported to have both OMS and SPS syndromes and an anti-Ri antibody. The anti-Ri antibody probably contributes to GABA autoreceptor dysfunction and causes a glutamate–GABA imbalance contributing to muscle spasm/stiffness [91]. Similarly, two cases of encephalomyelitis and SPS in association with breast carcinoma have been reported and were associated with anti-amphiphysin antibodies [92].

Another illustrative case was reported in which a female patient with clinical SPS was found to have a very high titer of anti-amphiphysin antibody (1:61,440). Based on the finding, she was diagnosed as having a paraneoplastic SPS, though no tumor could be found by routine mammography, CT scanning nor MRI. Eventually FDG-PET was performed and an axillary lymph node had FDG uptake. The lymph node was resected and it was found to contain metastatic breast adenocarcinoma. The patient was treated with steroids and chemotherapy and had a dramatic neurologic response [81, 93].

A rare complication of SPS is rhabdomyolysis. There is one reported case of breast cancer related SPS with a positive anti-amphiphysin titer and complicated by rhabdomyolysis. It is reported that SPS symptoms and rhabdomyolysis resolved with cancer therapy [94].

The electrophysiological characteristics of SPS are quite interesting. Typically SPS patients have continuous motor unit activity, co-contracting, and the presence of the cutaneo-muscular reflex. A Brazilian study of SPS suggested that patients with stiffness may display some or all of those three typical characteristics of SPS on electrophysiological analysis [95]. Expert neurology and oncology consultation is recommended for patients suspected to have SPS.

Finally, there have been a few suggestions for treatment of stubbornly refractory cases of SPS. One refractory case was treated with dantrolene [96]. Clonidine and tizanidine have been used historically. Other clinicians have used intrathecal ibuprofen [97–99] or spinal cord stimulators [100]. Tetrahydrocannabinol and cannabidiol as oromucosal sprays showed some symptomatic relief [101]. Physical therapy interventions with ultrasound, soft tissue mobilizations, manual stretching, and exercise have also been helpful. Physical therapy for stretching, joint mobility and gait retraining can last for years [100].

## Paraneoplastic neuropathy

One of the earliest available reports of a paraneoplastic sensorimotor neuropathy as a presenting symptom in breast cancer dates to 1994. That early report described nine patients with breast cancer with shared neurological manifestations of upper and lower muscle weakness, muscle cramps, paresthesia, numbness and radicular symptoms. The patients presented with the neurological manifestations up to 8 years before the discovery of the breast cancer [59]. Seven of the nine patients had tumor localized to the breast and the axillary lymph nodes and two had stage IV disease. The neurological manifestations were chronic, but disability was limited. Plasmapheresis was used in an attempt to relieve the neurological manifestations of the paraneoplastic syndrome, but only one patient improved transiently while on plasmapheresis. None of the nine patients in that early study were found to have detectable antibodies on CSF examination. Three of the patients improved with anti-cancer treatment [102].

Other literature reports in the breast cancer field include a report of a single case of paraneoplastic polyneuropathy published in 1997. In the report, a 59 year old woman was admitted complaining of numbness in all extremities and ataxia on the left. The patient was found to have breast cancer and had a left mastectomy and axillary lymph node resection. Sural nerve biopsy was performed and serum analysis detected the presence of an anti-neuronal antibody [36–38].

Two additional case reports were published in 2007 and 2014 with motor neuron dominant syndromes both of which improved significantly after the treatment of the breast cancer. One of the patients was treated with docetaxel and anastrozole and the other patient was treated with capecitabine. Another study of seven women with breast cancer and motor neuron disease also found cases of sensorimotor neuropathy preceding the diagnosis of breast cancer [103].

Research into sensorimotor neuropathies related to cancer have observed that anti-neuronal antibodies are detected in around 85 % of cases with the most common antibodies being anti-Hu, anti-Yo and anti-Ri [104, 105]. The absence of detectable antibody does not rule out a paraneoplastic process, as cell mediated nerve toxicity may act in the absence of humoral-mediated effect.

The patterns of paraneoplastic neurological manifestation may vary from patient to patient. In a review of 14 cases with paraneoplastic motor neuron disease, some patients had a rapidly progressive course and those were generally associated with anti-Hu antibodies. Other breast cancer patients with upper motor neuron disease and breast cancer had prolonged courses, no appreciable development of lower motor neuron signs, and no antibody detected in serum. The conclusion was that there may be wide variation in presentation and seropositivity in paraneoplastic neuropathy [104].

A recent retrospective study of 20 paraneoplastic neuropathy patients examined patterns of neuropathy in patients with anti-Hu antibodies. Nerve conduction assessments were performed and each nerve was classified as normal, demyelinating, axonal/neuronal or axonal/demyelinating. The study reported that, CNS neuropathy occurred in 40 %, autonomic neuropathy in 30 %, and peripheral neuropathy in 95 % of the patients (i.e. overlap existed). The course of progression of the disease differed between patients; it was acute in one patient (5 %), subacute in 55 %, and progressive in the rest of the study population (40 %). In the patients, peripheral neuropathy was reported to be sensory in 70 % of the patients and sensorimotor in 25 %. At onset, symptoms were symmetrical (65 %), asymmetrical (25 %) or multifocal (10 %). Clinically, the main presenting symptom was pain in 80 % of the patients. Nerve conduction revealed axonal/neuronal pattern to be the most frequent (46.9 % of studied nerves) while axonal/demyelinating or demyelinating patterns were seen in 18.3 and 4.9 % of nerves, respectively. The axonal/neuronal pattern was more frequent in sensory nerves and the mixed axonal/demyelinating pattern more frequent in motor nerves (p < 0.01). In patients with a clinically apparent sensory neuropathy, 88.5 % of sensory nerves were abnormal, mostly with an axonal/neuronal pattern. In patients with a sensorimotor neuropathy, 96.6 % of sensory nerves and 71 % of motor nerves were abnormal. The study concluded that pure sensory neuropathy is not a common paraneoplastic finding, while motor neuropathy may be a more frequent paraneoplastic effect [106].

There are no randomized controlled trials of treatment for paraneoplastic neuropathy to use for practice guidance. The evidence from case series, case reports and a few expert opinions would suggest that immunomodulation with IVIG, plasmapheresis, steroids or chemotherapy are likely to be useful.

## Paraneoplastic encephalomyelitis

Paraneoplastic encephalomyelitis is another rare paraneoplastic neurological manifestation of breast cancer. It may involve different areas of the CNS such as the hippocampus, the lower brain stem, spinal root ganglia or dorsal root ganglia [6, 38]. Due to involvement of variable areas of the nervous system, the clinical picture usually has a wide range of neurological manifestations. It can lead to limbic encephalitis (LE), brain stem syndromes, autonomic dysfunction, myelitis, chronic gastrointestinal pseudo-obstruction (CGP), cerebellar ataxia and sensory polyneuropathy (SSN). Limbic encephalitis, SSN and cerebellar ataxia are the most common clinical presentations and autonomic neuropathy is present in around 30 % of the patients [38, 107].

Specifically, LE is the most common form of paraneoplastic encephalomyelitis. It may present with an acute or sub-acute onset of symptoms such as confusion, loss of short term memory or seizures. In most reports, small cell lung cancer makes up the majority of cases of paraneoplastic LE, followed by testicular tumors in around 20 % of cases and then by breast cancer in 8 % of cases [5, 108, 109]. There are case reports of paraneoplastic limbic encephalitis involving the extra-limbic structures leading to overlap syndromes [110]. The diagnosis is most often made based on characteristic findings on a brain MRI. The findings of hyperintensity signals on T2-weighted or fluid attenuation inversion recovery (FLAIR) images involving one or both medial temporal lobes, when present, are specific for LE but they are not found in every patient [7]. The formal diagnosis of limbic encephalitis may be challenging. In one case series, there were 50 patients fulfilling criteria to be diagnosed with paraneoplastic limbic encephalitis, yet only 30 patients had anti-neuronal antibodies (18 anti-Hu, 10 anti-Ta, 2 anti-Ma) while 20 patients were anti-neuronal antibody negative. Half of the patients had signal abnormalities in the limbic system on MRI [7].

A well characterized example is reported for a patient with medullary breast cancer and paraneoplastic limbic encephalitis. The patient presented with clinical manifestations of limbic/brain stem encephalitis and anti-Ma2 antibodies in her serum and CSF (confirmed with western blot). The patient presented with multiple neurological signs that fit with the diagnosis of encephalitis. Both T2 weighted and FLAIR images on MRI showed areas of high signal strength in the bilateral mesial temporal lobes, the amygdala, the hippocampus, and the hypothalamus. There was no region of mass effect or gadolinium enhancement. The cerebral cortex and brain stem were not affected. A chest CT showed swelling of an axillary lymph node. A mammogram was ordered and showed a mass in the right breast that was not palpable. A needle biopsy was performed and medullary breast cancer was diagnosed. The cancer was excised but the neurological manifestations persisted and showed further deterioration. Intravenous methylprednisolone followed by oral prednisone were used and modest cognitive improvement was achieved, but the radiographic changes on the MRI were not reversible [39–44].

In regard to diagnosis, a study at Mayo clinic of LE patients used EEG and found focal or generalized slowing and/or epileptiform activity, maximal in the temporal regions, in all 22 patients assessed. MRI revealed increased T2 signal involving one or both temporal lobes in 15 of 18 patients. CSF results were abnormal in 18 of 23 patients tested. Clinical or radiographic evidence of extra-limbic involvement was documented in 12 of 22 patients [111].

Regarding treatment and follow up, the treatment of the tumor appeared to have a greater effect on the neurological outcome than did the use of immunosuppression. Improvement was observed in 38 % of anti-Hu patients, 30 % of anti-Ta patients and 64 % of patients without these antibodies [7]. In a series of 71 patients with paraneoplastic encephalomyelitis or sensory neuronopathy, treatment using steroids and plasmapharesis did not show improvement in any of the patients. In the studied patients, the most common cause of death was autonomic and respiratory failure, which was either of central origin or due to muscle weakness [112]. In a separate case series, with 14 % breast cancer patients, the only treatment that showed improvement or stabilization of the clinical condition was anti-tumor treatment [113]. Furthermore, a study examined the role of IVIG in 22 patients with paraneoplastic encephalomyelitis and sensory neuropathy syndromes. Ten patients in the study remained stable (eight patients with anti-Hu and two with anti-Yo antibodies), ten patients deteriorated and one patient had an initial stabilization response that worsened when the IVIG was stopped. No change in serum antibody titers was noticed with treatment with IVIG. The final study conclusion was that, IVIG use had little clinical benefit in patients with paraneoplastic encephalomyelitis [114].

Ultimately, controlling paraneoplastic encephalomyelitis is a challenging clinical problem. The patients are reported to have a poor quality of life and limited treatments as above. Therefore, symptom control is often the mainstay of therapy. Symptomatic treatment can include medications for seizure control and for autonomic symptom improvement. Also physiotherapy plays an important role in addition to occupational, speech and psychological therapies.

## Conclusion

Patients with neurologic manifestations that are unexplained by any other neurological disorder should be tested for antineural antibodies. Although the sensitivity and specificity of these antibodies are not 100 %, they can aide in the diagnosis. Likewise, their absence does not exclude the diagnosis of paraneoplastic syndromes as T cell mediated paraneoplastic syndromes are well reported. The immune mechanisms underlying these syndromes are partially explained by IFN-α and IL-12 production along with marrow resident B cell activation. It has been speculated that the impending addition of checkpoint inhibitors to the breast cancer armamentarium might increase the frequency of paraneoplastic syndromes in breast cancer, but the limited data to date has not demonstrated an increase in these syndromes. Nevertheless, investigators are urged to remain vigilant as this class of drugs is developed.

Owing to the rarity of these syndromes, few prospective studies are available and existing data is mostly based on observations. Treatment is often empiric and should be tailored for each case. In paraneoplastic neurologic syndromes, treatment of the underlying tumor is of vital importance. Secondarily, immunosuppressive therapy may be helpful in some cases (see Table 3). A moderate degree of therapy with steroids and/or IVIG, and/or plasma exchange may prove helpful, especially in OMS and paraneoplastic neuropathy. If more aggressive immunosuppression is deemed necessary, then cyclophosphamide, mycophenolate or rituximab may be considered. Immunosuppressive therapy should not be delayed as the sooner the treatment, the better the outcome. Evaluation for presence of auto-antibodies and cancer diagnosis may take some time, so treatment should be started based on clinical suspicion and after exclusion of infections and other causes [110, 115–117].

**Table 3:** Lists the best currently available treatment suggestions for each syndrome and references for the recommendation

Syndrome	Primary treatment	Secondary treatments	References
Paraneoplastic cerebellar degeneration	*Antitumor therapy* resection of the primary tumor increases the overall median survival rates *IVIG* IVIG may be used alone or in combination with secondary treatments	Cyclophosphamide	[45, 46]
		Methylprednisolone	[50, 51]
Opsiclonus–myoclonus syndrome	*Antitumor therapy* including surgery, chemotherapy, radiotherapy *Immunotherapy* (alternative) IVIG ± Corticosterods or Plasmapharesis	*Mycophenolate mofetil(MM)* Mycophenolate was found to be associated with decreased relapse rates after stopping immunotherapy *Symptomatic treatments* Benzodiazepines Valproic acid Gabapentin Baclofen Levetiracetam	[79, 118]
			[34, 119]
			[120, 121]
			[122]
Stiff person syndrome	*Antitumor treatment* alternatives: Benzodiazepines, IVIG, Plasmapharesis, Corticosteroids	Baclofen	[82, 88]
		Dantrolene	[89, 90]
		Clonidine	[91, 92]
		Tizanidine	[96–100]
		Physical therapy Intrathecal baclofen-if refractory	
Paraneoplastic limbic encephalomyelitis	*Antitumor treatment* *Symptomatic treatment* Physiotherapy Seizure control	*IVIG*	
		Corticosteroids	[7, 112]
		Plasmapharesis	[113, 114]
Paraneoplastic retinopathy	There are no controlled trials for the treatment of paraneoplastic retinopathy nor paraneoplastic peripheral neuropathy. The literature is deemed insufficient at this time to recommend management of these conditions. The referenced case series and case report suggest the use of IVIG, plasmapharesis, steroids and/or chemotherapy		[106, 123]
Paraneoplastic sensory peripheral neuropathy			

## Abbreviations

CDR: cerebellar degeneration related gene
CGP: chronic gastrointestinal pseudo-obstruction
CSF: cerebrospinal fluid
EEG: electroencephalogram
FLAIR: fluid attenuation inversion recovery
GAD: glutamic acid decarboxylase
IVIG: intravenous immunoglobulin
LE: limbic encephalitis
MRI: magnetic resonance imaging
OMS: opsoclonus–myoclonus syndrome
PCD: paraneoplastic cerebellar degeneration
SCLC: small cell lung cancer
SSN: sensory polyneuropathy
